# Loss of HSulf-1 promotes altered lipid metabolism in ovarian cancer

**DOI:** 10.1186/2049-3002-2-13

**Published:** 2014-08-18

**Authors:** Debarshi Roy, Susmita Mondal, Chen Wang, Xiaoping He, Ashwani Khurana, Shailendra Giri, Robert Hoffmann, Deok-Beom Jung, Sung H Kim, Eduardo N Chini, Juliana Camacho Periera, Clifford D Folmes, Andrea Mariani, Sean C Dowdy, Jamie N Bakkum-Gamez, Shaun M Riska, Ann L Oberg, Edward D Karoly, Lauren N Bell, Jeremy Chien, Viji Shridhar

**Affiliations:** 1 Department of Experimental Pathology, Mayo Clinic College of Medicine, Rochester, MN 55905, USA; 2 Division of Biomedical Statistics and Informatics, Mayo Clinic, Rochester, MN 55905, USA; 3 Henry Ford Health System, Detroit, MI 48202, USA; 4 Cancer Preventive Material Development Research Center (CPMRC), College of Oriental Medicine, Kyunghee University, Seoul 130-701, Republic of Korea; 5 Department of Anesthesiology, Mayo Clinic College of Medicine, Rochester, MN 55905, USA; 6 Department of Cardiovascular Disease, Mayo Clinic College of Medicine, Rochester, MN 55905, USA; 7 Department of Obstetrics and Gynecology, Mayo Clinic College of Medicine, Rochester, MN 55905, USA; 8 Metabolon, Inc, Durham, NC 27713, USA; 9 Department of Cancer Biology, University of Kansas Medical Center, Kansas City, KN 66160, USA

**Keywords:** HSulf-1, Lipogenesis, Lipolysis, Lipid droplets, Microarray and metabolite profiling

## Abstract

**Background:**

Loss of the endosulfatase HSulf-1 is common in ovarian cancer, upregulates heparin binding growth factor signaling and potentiates tumorigenesis and angiogenesis. However, metabolic differences between isogenic cells with and without HSulf-1 have not been characterized upon HSulf-1 suppression *in vitro*. Since growth factor signaling is closely tied to metabolic alterations, we determined the extent to which HSulf-1 loss affects cancer cell metabolism.

**Results:**

Ingenuity pathway analysis of gene expression in HSulf-1 shRNA-silenced cells (Sh1 and Sh2 cells) compared to non-targeted control shRNA cells (NTC cells) and subsequent Kyoto Encyclopedia of Genes and Genomics (KEGG) database analysis showed altered metabolic pathways with changes in the lipid metabolism as one of the major pathways altered inSh1 and 2 cells. Untargeted global metabolomic profiling in these isogenic cell lines identified approximately 338 metabolites using GC/MS and LC/MS/MS platforms. Knockdown of HSulf-1 in OV202 cells induced significant changes in 156 metabolites associated with several metabolic pathways including amino acid, lipids, and nucleotides. Loss of HSulf-1 promoted overall fatty acid synthesis leading to enhance the metabolite levels of long chain, branched, and essential fatty acids along with sphingolipids. Furthermore, HSulf-1 loss induced the expression of lipogenic genes including FASN, SREBF1, PPARγ, and PLA2G3 stimulated lipid droplet accumulation. Conversely, re-expression of HSulf-1 in Sh1 cells reduced the lipid droplet formation. Additionally, HSulf-1 also enhanced CPT1A and fatty acid oxidation and augmented the protein expression of key lipolytic enzymes such as MAGL, DAGLA, HSL, and ASCL1. Overall, these findings suggest that loss of HSulf-1 by concomitantly enhancing fatty acid synthesis and oxidation confers a lipogenic phenotype leading to the metabolic alterations associated with the progression of ovarian cancer.

**Conclusions:**

Taken together, these findings demonstrate that loss of HSulf-1 potentially contributes to the metabolic alterations associated with the progression of ovarian pathogenesis, specifically impacting the lipogenic phenotype of ovarian cancer cells that can be therapeutically targeted.

## Background

Metabolic re-programming has recently emerged as a new hallmark of cancer. Alteration of cellular metabolism in cancer cells is proposed to increase the availability of essential building blocks that support uncontrolled cellular proliferation [1]. Most cancer cells, although diversified by etiology and type, reprogram their metabolism to accumulate metabolic intermediates as sources of building blocks [2]. The Warburg effect is one of the most important metabolic alteration in cancer, in which neoplastic cells exhibit higher glucose uptake and utilization by altering glucose metabolism even in the presence of oxygen to produce lactate from glucose and thereby decouples glycolysis from mitochondrial oxidation [3]. Although alterations in fatty acids (FAs) and lipid metabolism have received less attention, recently, their importance in cancer metabolism is being increasingly recognized. The total lipid pools required for membrane synthesis of dividing cells are derived mainly from FAs and in part from acetyl CoA [4]. Cancer cells meet their FAs demand mainly by increasing *de novo* FA synthesis rather than from exogenous sources and partly from acetyl CoA [5,6]. The activated FAs can then be utilized in the synthesis of membrane phospholipids (PLs) including phosphatidylcholine (PC), phosphatidylethanolamine (PE) in addition to sterols, sphingolipids, and lysolipids to meet the energy demands and proliferation. The rest of the activated FAs then function as signaling molecules or esterified as triglycerols or sterol esters and stored in lipid droplets (LDs) [7,8].

Accumulating evidence suggest that activation of oncogenes such as *MYC*, NF-kB, *K-RAS*[2,9] and loss of tumor suppressor genes (*P53*, *LKB1/AMPK*) [10-12] forms a basis for altered metabolism of cancer cells. The oncogenic activation of c-MYC turns on the Ras-Raf-MAPK signaling pathway along with HIF1α and PI3K-Akt-mTOR axis which transcriptionally stimulate the expression of most glycolytic and glutaminolytic genes and subsequently activate lipid metabolism [13]. Similarly, loss of tumor suppressors forms the basis of the Warburg effect leading to carcinogenesis. For example, p53 activates TIGAR to reduce the cellular accumulation of fructose 2,6 bisphosphate, an allosteric activator of phosphofructo kinase, a critical control point in glycolysis leads to decreased glycolysis [10]. Moreover, growth factor-mediated phosphorylation of pyruvate kinase isoform 2 (PKM2) [14] and mutations of genes in IDH1/2 [15] in the metabolic pathways such as glycolysis and the TCA cycle respectively have been identified that support growth of the rapidly proliferating cells and survive metabolic stress [16,17].

Human Sulfatase 1 (HSulf-1), an endosulfatase established as a putative tumor suppressor in ovarian cancer, has been shown to modulate the signaling of growth factors and cytokines in tumor microenvironment [18]. Our previous work demonstrated that loss of HSulf-1 modulates heparin-binding growth factors such as bFGF, VEGF, HGF, PDGF, and heparin binding EGF (HB-EGF) signaling, which plays an important role in tumor progression, metastasis, and angiogenesis [19-22]. Moreover, serous tumors with moderate to high levels of HSulf-1 had better prognosis in terms of overall survival, implicating its critical role in the progression of ovarian cancer [23]. Our more recent data demonstrated that HSulf-1 knockdown clones in the OV202 ovarian cancer cell line (OV202Sh1 and Sh2 cells) have significantly increased the ability to form anchorage-independent colonies *in vitro* and enhanced tumorigenicity *in vivo*[24]. Also, in breast cancer, HSulf-1 is negatively regulated by HIF1α, but positively by von Hippel-Lindau tumor suppressor gene [23,25,26]. These findings led us to hypothesize that HSulf-1 might play a unique role to alter tumor microenvironment raising the possibility that its loss might alter the cellular metabolism and levels of the resulting metabolites as a downstream effect of altered growth factor signaling.

In the current study, global changes in metabolism were investigated in HSulf-1 silenced OV202 cells by microarray, metabolic data analysis, and Western blotting. Here, we for the first time report that loss of HSulf-1 promotes overall fatty acid synthesis and oxidation leading to a lipogenic phenotype to promote cancer growth in ovarian cancer.

## Methods

### Cell culture

OV202 cell line was low-passage primary line established at the Mayo Clinic [27]. OV202NTC, Sh1, Sh2, and Cl 11 cells were cultured in 5% CO_2_-95% air humidified atmosphere at 37°C with minimal essential medium supplemented with 20% fetal bovine serum and 1 μg/ml puromycin, with nonessential amino acids. All cell lines were tested using a PCR-based assay and found to be free of Mycoplasma contamination.

### ShRNA

HSulf-1 short-hairpin (sh) RNA1 (Sh1- AGCTACCCTGGGTTCCTTTGT) which targets the 3′-untranslated region (UTR) was cloned into lentiviral vector pLKO.1-puro as described previously [27]. HSulf-1 shRNA2 (Sh2- CGTCGAATTTGAAGGTGAAAT) and nontargeted control shRNAs (NTC shRNA- ACTTACGAGTGACAGTAGATT) cloned into the lentivirus vector pLKO.1-puro were chosen from the human library (MISSION TRC-Hs 1.0) and purchased as glycerol stock from Sigma. Transfection with Fugene (Roche) was performed according to the manufacturer's instructions. Transducted cells were selected with 1 μg/ml puromycin.

### Rescue of HSulf-1 in Sh1 cells

pcDNA-HSulf-1 plasmid was cloned as described previously [19]. Since Sh1 shRNA targeted the 3′UTR of HSulf-1, we rescued the expression of HSulf-1 in this cell line with CMV-driven WT expression construct and selected stable clone C11 as previously described [19]. Vector only transfected cells served as controls.

### Microarray expression data analysis

OV202 NTC, Sh1, and Sh2 cells in triplicates were profiled using Illumina Human HT-12 3.0 Expression Beadchip array as previously described [28]. Microarray expression data were analyzed on the log2 scale. Data quality was assessed via box and whisker plots along with residual and pair-wise MVA plots before and after normalization [29,30]. All arrays were normalized together using fastlo, a non-linear normalization similar to cyclic loess which runs in a fraction of the time [31]. Both supervised and unsupervised analyses were performed. Supervised analysis to determine differentially expressed genes was performed using Significance Analysis of Microarrays (SAM) [32]. For SAM analysis for unpaired samples, Biometric Research Branch (BRB)-ArrayTools (Version 3.7.0, developed by Dr. Richard Simon and Amy Peng Lam.) was used with Delta set to 0.822, resulting in false discovery rate <5%. Unsupervised clustering was performed using the one minus correlation metric with average linkage. Heat maps were generated for visualization. Pathway analysis was performed using Ingenuity Pathway Analysis (Ingenuity® Systems, http://www.ingenuity.com).

### Liquid chromatography/mass spectrometry (LC/MS, LC/MS^2^)

The LC/MS portion of the platform was based on a Waters ACQUITY UPLC and a Thermo-Finnigan LTQ mass spectrometer, which consisted of an electrospray ionization (ESI) source and linear ion-trap (LIT) mass analyzer. The sample extract was split into 2 aliquots, dried, then reconstituted in acidic or basic LC-compatible solvents, each of which contained 11 or more injection standards at fixed concentrations. One aliquot was analyzed using acidic positive ion optimized conditions and the other using basic negative ion optimized conditions in two independent injections using separate dedicated columns. Extracts reconstituted in acidic conditions were gradient eluted using water and methanol both containing 0.1% Formic acid, while the basic extracts, which also used water/methanol, contained 6.5 mM ammonium bicarbonate. The MS analysis alternated between MS and data-dependent MS^2^ scans using dynamic exclusion.

### Gas chromatography/mass spectrometry (GC/MS)

The samples destined for GC/MS analysis were re-dried under vacuum desiccation for a minimum of 24 h prior to being derivatized under dried nitrogen using bistrimethyl-silyl-triflouroacetamide (BSTFA). The GC column was 5% phenyldimethyl silicone and the temperature ramp is from 40°C to 300°C in a 16-min period. Samples were analyzed by a Thermo-Finnigan Trace DSQ fast-scanning single-quadrupole mass spectrometer using electron impact ionization. The instrument was tuned and calibrated for mass resolution and mass accuracy on a daily basis. The information output from the raw data files was automatically extracted as discussed below.

### Data extraction and compound identification

Peaks were identified using Metabolon's proprietary peak integration software. Compounds were identified by comparison to library entries of purified standards or recurrent unknown entities. Identification of known chemical entities was based on comparison to metabolomic library entries of purified standards. As of this writing, more than 2,600 commercially available purified standard compounds had been identified and registered into LIMS for distribution to both the LC and GC platforms for determination of their analytical characteristics. The combination of chromatographic properties and mass spectra gave an indication of a match to the specific compound or an isobaric entity. Metabolon data analysts use proprietary visualization and interpretation software to confirm the consistency of peak identification among the various samples. Library matches for each compound were checked for each sample and corrected if necessary.

### Normalization

Raw data from each sample was normalized to protein concentration as measured by Bradford assay prior to statistical analysis.

### Sample accessioning

Each sample received was accessioned into the Metabolon LIMS system and was assigned by the LIMS, a unique identifier, which was associated with the original source identifier only. This identifier was used to track all sample handling, tasks, and results*.* The samples (and all derived aliquots) were bar-coded and tracked by the LIMS system. All portions of any sample were automatically assigned their own unique identifiers by the LIMS when a new task was created; the relationship of these samples was also tracked. All samples were maintained at -80°C until processed.

### Sample preparation

The sample preparation process was carried out using the automated MicroLab STAR® system from Hamilton Company (Reno, NV, USA). Recovery standards were added prior to the first step in the extraction process for QC purposes. Sample preparation was conducted using a proprietary series of organic and aqueous extractions to remove the protein fraction while allowing maximum recovery of small molecules. The resulting extract was divided into four fractions; two for analysis by LC and one for analysis by GC and a forth as a spare. Samples were placed briefly on a TurboVap® (Zymark, Hopkinton, MA, USA) to remove the organic solvent. Each sample was then frozen and dried under vacuum. Samples were then prepared for the appropriate instrument, either LC/MS or GC/MS.

### QA/QC

For QA/QC purposes, a number of additional samples are included with each day's analysis. Furthermore, a selection of QC compounds is added to every sample, including those under test. These compounds are carefully chosen so as not to interfere with the measurement of the endogenous compounds. These QC samples are primarily used to evaluate the process control for each study as well as aiding in the data curation.

### Metaboanalyst

Differently expressed metabolites between Sh1/Sh2 and baseline conditions were firstly mapped to KEGG metabolites IDs according to Human Metabolome Database (HMDB; URL: http://www.hmdb.ca/) [33]. Then, pathway analysis was performed to highlight relevant metabolic pathways defined in KEGG database ( http://www.genome.jp/kegg/), using an on-line tool named MetaboAnalyst ( http://www.metaboanalyst.ca/) [34,35]. Specifically, two types of pathway analysis were done: one is over-representation analysis using hypergeometric test [35], asking if differentially expressed metabolites are particularly enriched in a same pathway; the other is pathway topology analysis summarizing relative-betweeness centrality [34], investigating potential pathway impact of observed metabolite changes based on known pathway topology relationships.

### Western blot analysis

Western blot analysis was performed as described previously [36]. Whole cell lysates were analyzed with the following antibodies: FASN, ASCL1 (Cell signaling), SREBP1c, PLA2G3, HSulf-1 (Abcam, AB96533), CPT1A, HSL, DAGLA, β-tubulin (GeneTex) and β-actin (Sigma-Aldrich).

### Real-time PCR

Quantitative real-time PCR (qRT-PCR) was carried out using SYBR-Green PCR Master Mix (Applied Biosystems, Foster City, CA, USA), with specific primers for the genes shown in this study. GAPDH or 18S ribosomal subunit (Applied Biosystems) were used as internal control in a Light Cycler kit (BioRad Chromo 4). Normalization across samples was carried out using the average of the constitutive human gene 18S and/or GAPDH primers and calculated as previously described [18]. Binding efficiencies of primer sets for both target and reference genes were similar.

### Bodipy staining

Cells (50,000) were seeded on a coverslip in a 24-well plate and were grown for 24 hours in the presence of complete growth medium. Cells were washed and fixed in 4% paraformaldehyde for 10 min at room temperature before staining with 1 μg/ml BODIPY (493/503; Sigma, St. Louis, MI, USA) in PBS for 10 min at room temperature. Coverslips were washed with PBS and mounted in a slide with Prolong Gold Antifade Reagent (Invitrogen). BODIPY stained cells were examined under inverted confocal fluorescence microscope (Zeiss).

### Transient transfection

To determine the effect of PLA2G3 on lipid droplet biogenesis OV202 NTC cells were transiently transfected with plasmids containing empty vector or cDNA encoding PLA2G3. After 24 h of transfection, we performed BODIPY staining to visualize lipid droplets. PLA2G3 plasmid was obtained on a MTA from Addgene.

### Fatty acid synthesis

Cells were washed twice in cold PBS and resuspend in lysis buffer (50 mM Tris-HCl, pH 7.4, 1 mM EDTA, 150 mM NaCl and PMSF. Cells were sonicated and homogenized by dounce homogenizer followed by centrifugation at 13,000 rpm for 15 min at 4°C. The supernatant was collected and proteins were measured by Bradford assay, and 100 μg of protein was used to conduct FASN activity assay. FASN activity was measured by protocol described by Vazquez-Martin et al. [37]. Briefly, 100 μg of protein was incubated with 240 μM NADPH, 30 μM acetyl CoA and 50 μM malonyl CoA in assay buffer (200 mM potassium phosphate, pH 6.6, 1 mM DTT, 1 mM EDTA) and oxidation of NADPH was measured by monitoring the absorbance at 340 nM over the period of the time. Results presented here compares FASN activity monitored for 10 min. Values are presented as nanomolar NADPH oxidized per minute per milligram of protein.

### Fatty acid oxidation

Oxygen consumption rate was measured using a Seahorse Bioscience XF24 flux analyzer. 5 × 10^4^ cells were seeded per well in triplicates in MEM-α containing 20% FBS in an XF24 well culture microplates and incubated overnight in a 37°C/10% CO_2_ incubator. The assay medium for FAO is low-buffered KHB buffer (110 mM NaCl, 4.7 mM KCl, 2 mM MgSO_4_, 1.2 mM Na_2_HPO_4_, 2.5 mM glucose adjusted to pH 7.4) supplemented with 0.5 mM carnitine. For induction of FAO, BSA conjugated palmitate was injected to a final concentration of 50 μM. XF analyses were performed in the XF Extracellular Flux Analyzer (Seahorse Bioscience, Billerica, MA, USA). Three basal rates were measured prior to automated injection of palmitic acid (50 μM) coupled to BSA vehicle or BSA vehicle alone. After treatment for 55 min, the carnitine palmitoyl transferase-1 inhibitor, Etomoxir (ETO, 50 μM), was added. Oxygen consumption rates were measured by using time-resolved method (Seahorse Bioscience XF24) (21). Data were normalized to protein content (assayed after completion of measurements).

### Proliferation assay

Equal number of cells (1 × 10^5^) was plated in triplicate in 12-well plates. OV202NTC, Sh1, and Sh2 cells were counted after 24, 48, and 72 h using a cellometer (Nexelom, Lawrence, MA, USA). For Etomoxir treatment, Equal number of cells (1 × 10^5^) were seeded in 12-well plates in triplicate and treated with increasing concentration of Etomoxir (0 to 100 μM) for 24 h and total cell numbers were counted using cellometer.

## Results

### Loss of HSulf-1 comprehensively altered major metabolic pathways

**Figure 1:**
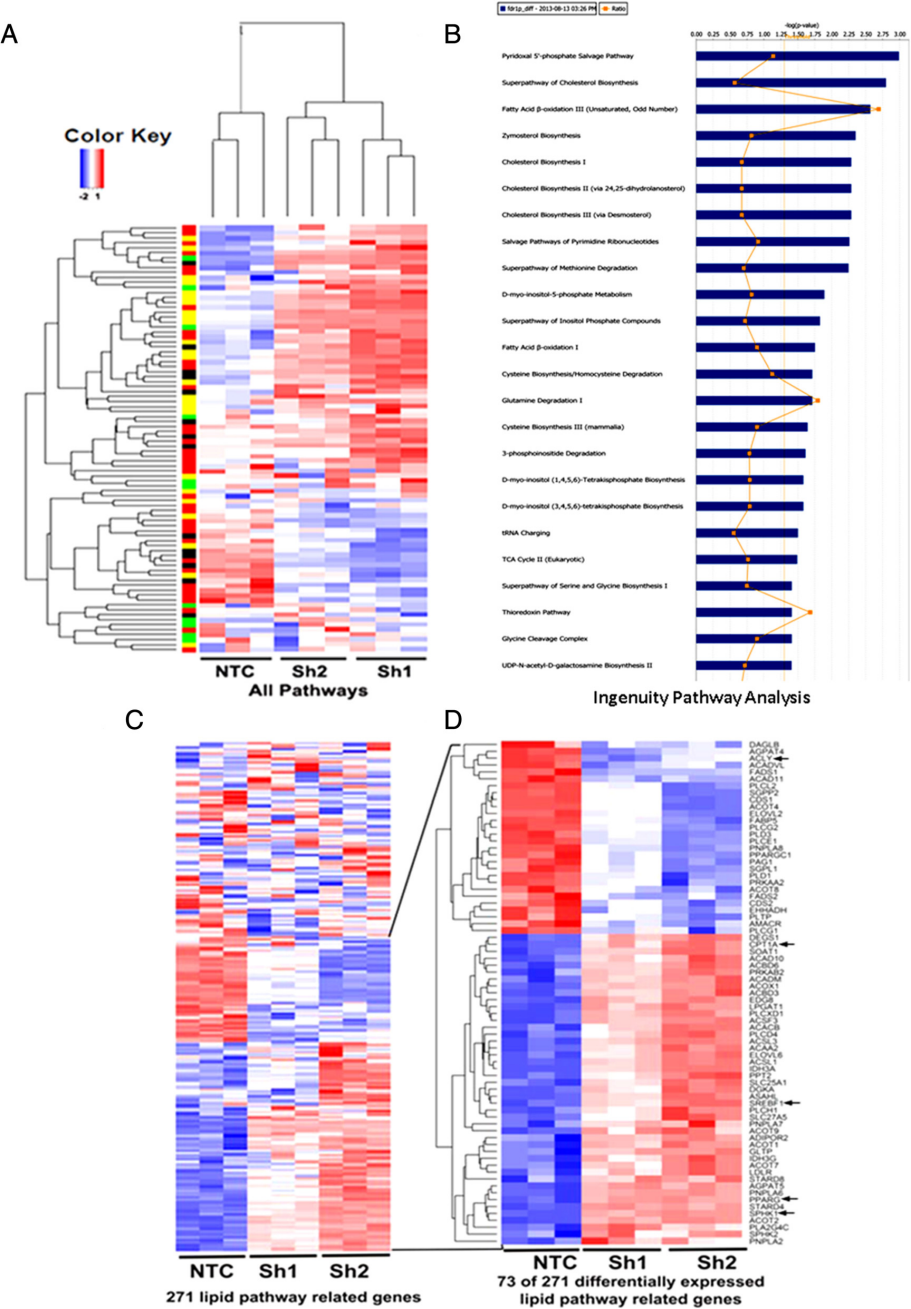
**Microarray analysis of differentially expressed genes in OV202NTC, Sh1, and Sh2 cells. (A)** Unsupervised hierarchical clustering normalized expression values of 20,090 selected probe sets for NTC, Sh1, and Sh2. Each class is represented by three biological replicates. *Red*: expression values above the average across all samples; *blue*: expression values below the average across all samples. **(B)** Ingenuity pathway analysis of metabolic genes. The most statistically significant metabolic pathways identified in the confirmed cell-specific marker list are listed according to their *p* value (-Log) (*blue bars*) and the ratio of list genes found in each pathway over the total number of genes in that pathway (Ratio, orange squares). The threshold line corresponds to a pathway enrichment *p* value of 0.05. **(C)** Unsupervised hierarchical clustering normalized expression values of 271 selected lipid pathway related probe sets for NTC, Sh1, and Sh2. Each class is represented by three biological replicates. *Red*: expression values above the average across all samples; *blue*: expression values below the average across all samples. **(D)** The most differentially expressed lipid pathway related genes (73 of the 271 genes in A, FDR = <1%). The statistical difference was tested according to *t* test using ‘genefilter’ package in R, and multi hypothesis-testing corrected FDR was estimated using ‘fdrtool’ package in R. The *black arrow* indicates genes analyzed by real-time and/or western blot analysis in this study.

We recently reported that HSulf-1 knockdown clones in the OV202 ovarian cancer cell line (OV202Sh1 and Sh2 cells) have significantly increased ability to form anchorage-independent colonies *in vitro* and enhanced tumorigenicity *in vivo*[24]. Consistent with these observations, our growth assays showed enhanced growth rate in Sh1 and Sh2 cells compared to NTC cells (Additional file 1: Figure S1). Here, to elucidate the function of putative tumor suppressor HSulf-1 in the metabolism of ovarian cancer, we performed gene expression profiling of stably knockdown HSulf-1 clonal lines OV202 Sh1 and Sh2 cells (referred to from hereon as Sh1 and Sh2) compared to HSulf-1 expressing non-targeted control cells (OV202NTC, referred to as NTC) [26] in triplicates using Illumina HumanHT 12 v3 platform [28]. Unbiased hierarchical clustering and heat maps showed that genes in several different pathways were differentially expressed in Sh1 and Sh2 compared to NTC cells (Figure 1A). We found that over 1,645 and 780 genes were differentially expressed in Sh1 and Sh2 cells, respectively, compared to NTC at 2.6 FC (*p* and FDR <0.0001). We identified 500 and 280 altered genes in Sh1 and Sh2 cells respectively by significance analysis of microarrays (SAM) [32] from the comprehensive list of 2,752 genes which encoded all known human metabolic enzymes and transporters reported by Possemato et al. [38]. Ingenuity pathway analysis ( http://www.ingenuity.com) for these genes showed that most genes were differentially regulated in the fatty acid/lipid pathways in Sh1 and Sh2 cells compared to NTC cells (Additional file 2: Table S1, Figure 1B). We next explored the 271 genes in the lipid related pathways from our microarray data by unsupervised clustering (Figure 1C) and found that 26% (73 of 271) genes were differentially expressed in Sh1 and Sh2 cells compared to NTC cells (Figure 1D), indicating that tumor suppressor HSulf-1 possibly regulates the lipid metabolism in ovarian cancer cells.

### Loss of HSulf-1 altered global metabolic profile in ovarian cancer cells

**Figure 2:**
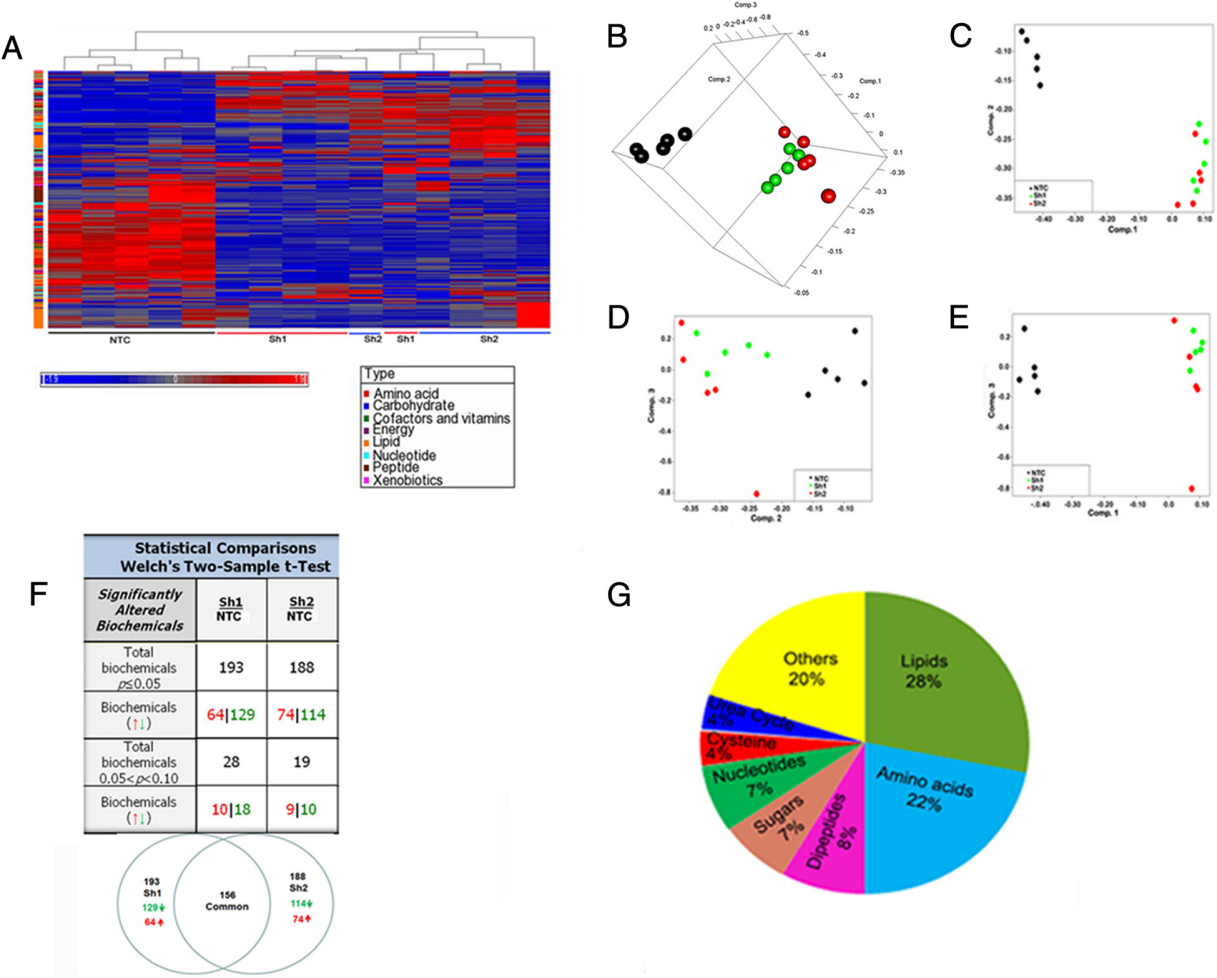
**Metabolite profile of ovarian cell line expressing HSulf-1 (NTC) and HSulf-1 downregulates Sh1 and Sh2 cells. (A)** Heatmap showing 338 biochemicals in lysates from 5 replicates each of OV202 cells expressing HSulf-1 (NTC), HSulf-1 Sh1 and Sh2 cells arranged by unsupervised clustering. **(B)** Three-dimensional sample PCA plot of log-transformed normalized concentration of 338 biochemicals, where samples were colored by NTC, Sh1, and Sh2 cell-types. **(C, D, E)** 2-D PCA plots: two-dimensional PCA plots between paired principal components (first PC vs. second PC; second PC vs. third PC; first PC vs. third PC). *x*-/*y*-axis corresponds to loadings of specified PC dimension and each point is a metabolite sample, with *black*, *green*, and *red colors* indicating NTC, Sh1, and Sh2 conditions, respectively. **(F)** Table showing significantly altered biochemical in Sh1 and Sh2 cells compared to NTC cells. Welch's two-sample *t* test was used to identify biochemicals that differed significantly between experimental groups. A summary of the numbers of biochemicals that achieved statistical significance (*p* ≤ 0.05), as well as those approaching significance (0.05 < *p* < 0.10), are shown along with the Venn diagram of shared metabolites between Sh1 and Sh2 cells. **(G)** Pie chart of percentage of common altered metabolites in Sh1 and Sh2 cells in major pathways.

To determine whether loss of HSulf-1 has effect in cellular metabolism, we performed unbiased global metabolic profiling using the Metabolon platform (Metabolon Inc, Durham, NC, USA) in Sh1 and Sh2 cells compared to NTC. The samples were extracted using Metabolon’s standard solvent extraction method from cells in logarithmic phase with 5 biological replicates for each sample and distributed into equal parts for analysis on the GC/MS and LC/MS/MS platforms. Hierarchical clustering revealed that metabolite levels of Sh1 and Sh2 cells cluster together separately from control NTC (Figure 2A). Our initial principal component analysis (PCA) [39] revealed that Sh1 and Sh2 cells had specific group of metabolites which were different from NTC cells (Figure 2B). Additionally, *t* test in each principal component dimension showed that PCA differences between Sh1-Sh2 and NTC cells mainly reside in the first and second principal components, with statistically significant *p* values of 1.8e - 10 and 4.5e - 6, respectively. Consistent with this, two-dimensional PCA plots showed differences between Sh1-Sh2 and NTC are mainly in the first and second PC dimensions (Figure 2C,D,E). We also performed linear regression analysis to evaluate association of each principal component vs. major metabolite classes, to determine if lipid class is one of the dominant factors determining the first and second principal components. The association was evaluated in a multivariate regression model (Additional file 3: Figure S2), where coefficients of first/second PCs were treated as dependent variable, and metabolite class labels were regarded as independent variables. This analysis showed that the lipid class has statistically significant association with both first and second PC dimensions, with *p* values = 5.0e - 3 and 1.4e - 3, respectively. Interestingly, the peptide class, despite its relatively small size, also has significant associations with both first and second PC dimensions, while the amino acid class has significant association with the first principal component.

Moreover, this analysis showed a total of 338 known metabolites altered by the loss of HSulf-1 in Sh1 and Sh2 cells. Among them, Sh1 and Sh2 cells had a total of 193 and 188 biochemical, respectively, which were significantly altered (*p* < 0.05, Welch's *t* test) compared to NTC cells (Figure 2F, Additional file 4: Table S2, and Additional file 5: Table S3). Additionally, with the loss of HSulf-1, 129 and 114 metabolites were downregulated, whereas 64 and 74 metabolites were upregulated in Sh1 and Sh2 cells, respectively. Also, 40% of these biochemicals were altered in the same direction in Sh1 and Sh2 cells compared to NTC cells (*p* = <0.05, with false discovery rate (FDR) <0.05, Additional file 6: Table S4). Interestingly, we found that among all the metabolites altered, 28% were lipids, 22% were amino acids, whereas sugar and dipeptide comprised 7% (Figure 2G), implicating that loss of HSulf-1 mediates a global metabolic alteration in ovarian cancer with changes in the lipid class being a major contributor.

However, some metabolites were differentially altered in the Sh1 and Sh2 cells. The differentially changed metabolites between these ShRNAs could also be a function of the level of knockdown and where the ShRNAs could have integrated. The extent of knockdown in Sh1 targeting the 3'UTR was close to 100%. However, with Sh2 RNA targeting the open reading frame, there was still some level of HSulf-1 present (Figure 1A, [24]).

### Alteration of lipid metabolites upon HSulf-1 loss

**Figure 3:**
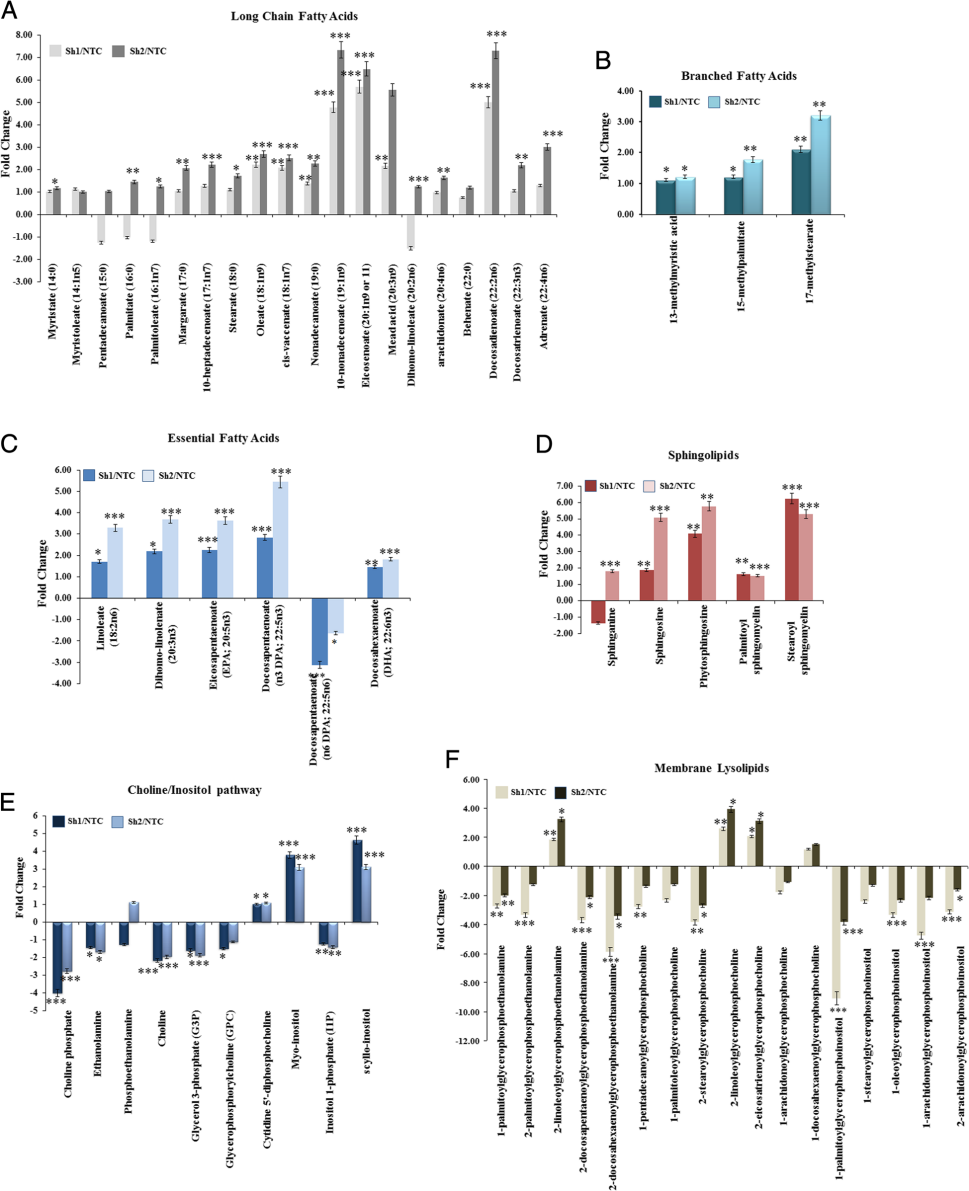
**Loss of HSulf-1-mediated increase in fatty acids, sphingolipids, and lysolipids.** The samples were extracted using Metabolon's standard solvent extraction method from cells in logarithmic phase having five biological replicates for each sample and distributed into equal parts for analysis on the GC/MS and LC/MS/MS platforms. **(A)** Fold increase of long-chain fatty acids were calculated by the average metabolite level of Sh1/NTC and Sh2/NTC. *p = 0.02 to 0.09; **p = 0.002 to 0.01; ***p < 0.001 compared to NTC. **(B)** Branched chain fatty acids and (**C**) essential fatty acids were calculated by the average metabolite level of Sh1/NTC and Sh2/NTC. *p* values for both branched chain fatty acids and essential fatty acids were *p = 0.01 to 0.02; **p = 0.002 to 0.01; ***p < 0.001 compared to NTC. **(D)** Fold increase of sphingolipids were calculated by the average metabolite level of Sh1/NTC and Sh2/NTC. *p = 0.01 to 0.09; **p = 0.002 to 0.008; ***p < 0.001 compared to NTC. **(E)** Choline/inositol pathway metabolites' fold change and **(F)** membrane lysolipids were calculated as mentioned earlier. *P* values for choline/inositol pathway was *p = 0.009 to 0.09; **p = 0.002 to 0.008; ***p < 0.001 whereas *p = 0.01 to 0.02; **p = 0.002 to 0.009; ***p < 0.001 for membrane lysolipids compared to NTC.

The lipogenic phenotype characterized by the activation of lipid metabolism is recognized as a universal feature of most cancers [40,41]. Apart from the fatty acid (FA) uptake, cancer cells requires de novo FA biosynthesis to synthesize new membranes, to store energy in lipid droplets and to form the lipidic platform for signaling in membrane level in lipid rafts for increased signaling of cell growth receptors [42,43]. Moreover, circulating lipids also play a significant role in cancer cell growth, migration, and invasion [44,45]. Of the 156 common metabolites altered in Sh1 and Sh2 cells, 44 (28%) metabolites belonged to the lipid class including long-chain FAs, lysolipids, sphingolipids, glycerolipids, eicosanoids, and carnitine (Additional file 6: Table S4), in which 20 metabolites were upregulated (45%). Using the Kyoto Encyclopedia of Genes and Genomics (KEGG) database, we mapped these metabolites to major pathways impacted with alterations in the key junctions [34,35]. These included linoleic acid, glycerophospholipid, arachidonic acid (Additional file 7: Figure S3), and sphingolipid pathways [46,47]. Consistent with these data, we found that among the 20 long-chain fatty acid metabolites detected, 13 metabolites were significantly increased by 2–6-fold including palmitate, stearate, and oleate, while the remaining 7 long-chain FAs did not show any significant differences in both Sh1 and Sh2 cells (Figure 3A). Of note, docosadienoate (22:2n6), 10-nonadeconoate and eicosenoate (20:1n9) levels were increased approximately sevenfold compared to NTC. Additionally, all the detected branched fatty acid metabolites were augmented (Figure 3B) while five of six essential fatty acids detected were increased 2–5.5-fold (Figure 3C).

The key structural lipids in cell membranes are the glycerol-phospholipids including phosphatidyl-choline, phosphatidyl-ethanolamine, phosphatidyl-serine, phosphatidyl-inositol, and phosphatidic acid in addition to other lipids, such as sterols, sphingolipids, and lyso-phospholipids. Sphingolipids such as ceramide, sphingosine, and sphingosine-1-phosphate are bioactive lipids which can dictate the signaling including growth factor responses, inflammation, apoptosis, and proliferation [48]. Our metabolite analysis revealed that HSulf-1 deficiency was closely associated with increased levels of sphinganine (FC = 21.79, *p = <0.001*), sphingosine (FC = 5.08, *p = <0.001*), palmitoyl sphingomyelin (FC = 1.62, *p = <0.0047*, and FC = 1.52, *p = <0.001*), and stearoyl sphingomyelin (FC = 6.24, *p = <0.001* and FC = 5.30, *p = <0.001*) (Figure 3D). Additionally, we found that knockdown of HSulf-1 expression also largely affected the metabolite levels of choline/inositol pathway (Figure 3E) and lysophospholipids (Figure 3F) including reduced glycerophosphorylcholine (GPC) (FC = -1.5, *p = 0.0940* in Sh1 cells only) and glycerol 3-phosphate (G3P) (FC = -1.6, *p = 0.0154* and FC = -1.9, *p = <0.001*). Altogether, these results implicated a key role of HSulf-1 in increased lipid metabolism and signaling.

### Deficiency of HSulf-1 in ovarian cancer induced higher expression of ‘lipogenic genes’

**Figure 4:**
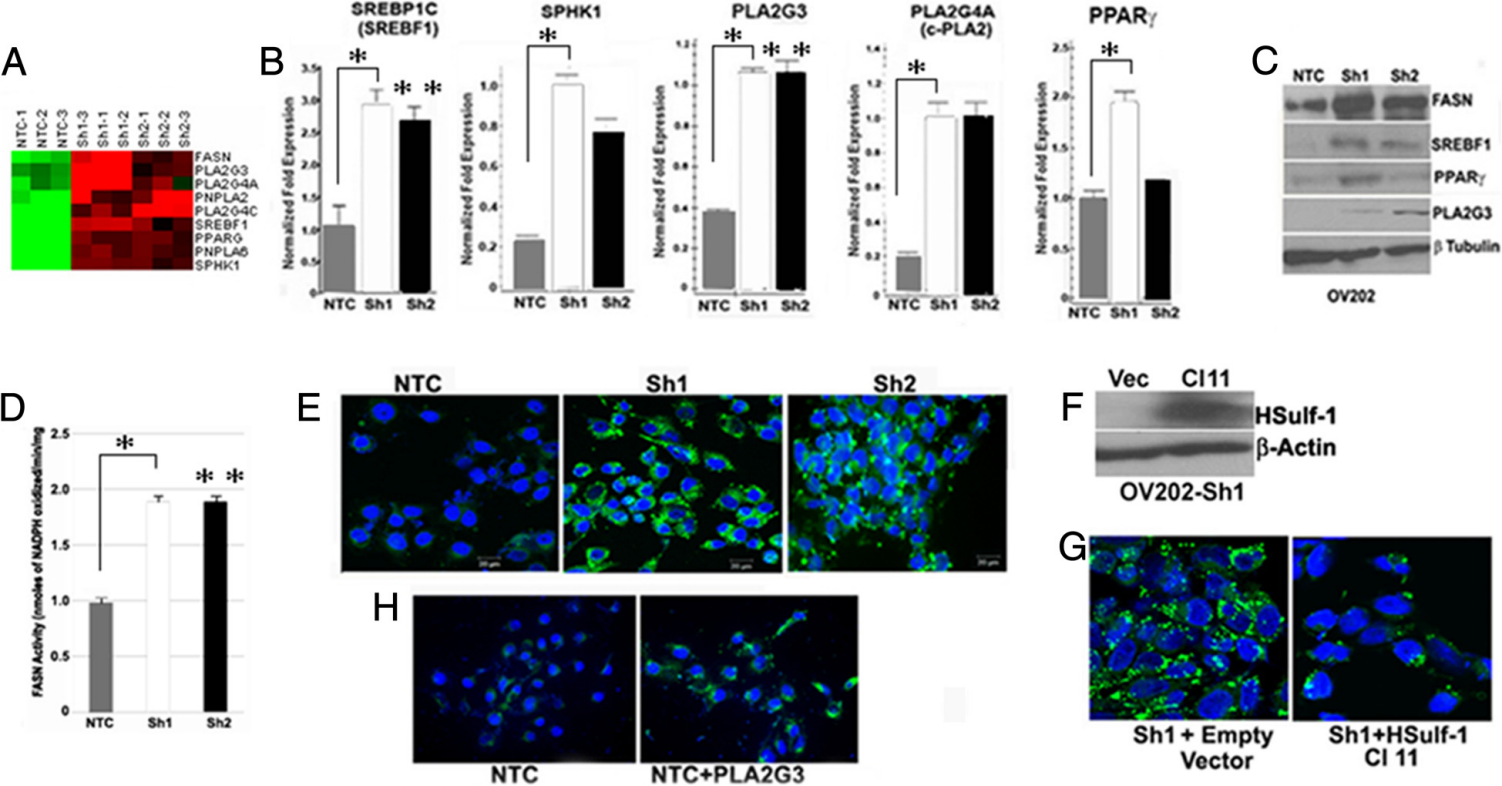
**Enhanced expression of lipogenic genes in ovarian cancer. (A)** Heat map of a subset of significantly altered lipid pathway-related genes by supervised clustering (*Red*, overexpressed and *green*, downregulated genes in Sh1 and Sh2 compared to NTC cells). **(B)** Normalized levels of *SREBP1c* (*SREBF1*), *SPHK1*, *PLA2G3*, *PLA2G4A* (*c-PLA2*) and PPARγ mRNA by quantitative RT-PCR in NTC and Sh1 and Sh2 cells. *p < 0.05; **p < 0.01; ***p < 0.001 compared to NTC cells. **(C)** Western blot analysis of FASN, SREBF1, PPARγ, PLA2G3 with beta tubulin as loading control. **(D)** FASN activity is expressed as fold change in Sh1 and Sh2 cells compared to NTC cells. Results are means (*columns*) of two independent experiments made in triplicate. One-factor ANOVA was used to analyze the differences in FASN activity between each experimental condition. All statistical tests were two-sided (**p ≤ 0.01). **(E)** Bodipy (*green*) and DAPI (*blue*) staining of the lipid droplets in NTC, Sh1, and Sh2 cells imaged in Carl Zeiss LSM 510S confocal microscope. **(F)** Immunoblot analysis of HSulf-1 in Sh1 vec, and Sh1 Cl 11, where β-actin was used as loading control. **(G)** Bodipy (*green*) and DAPI (*blue*) staining of lipid droplets and nuclei respectively in Sh1 vec and Sh1 Cl 11 cells. **(H)** Bodipy staining of LDs following enhanced expression of empty vector compared to PLA2G3 construct by transient transfection in OV202NTC cells shows LDs only in PLA2G3 transfected cells.

All the lipid molecules in cells are derived in part from acetyl CoA, and many contain FAs. These FA building blocks come from either exogenous sources or from de novo FA synthesis. Thus, malignant cells synthesize their own FA de novo and thereby exhibit a preference over exogenous FA uptake, while most normal human cells prefer exogenous sources [49]. Our microarray analysis showed that mRNA levels of lipogenic enzymes fatty acid synthase (*FASN),* sphingosine kinase 1 (*SPHK1*)*, PLA2G4A, PLA2G3,* sterol regulatory element-binding transcription factor 1 (*SREBF1*), and peroxisome proliferator-activated receptor (*PPARγ*) are also upregulated upon loss of HSulf-1 (Figure 4A). Consistent with the increased levels of these genes and lipid metabolites, qRT PCR and immunoblotting showed enhanced mRNA (Figure 4B) and protein (Figure 4C) expressions of *FASN*, *SPHK1*, *PLA2G4A*, *PLA2G3*, *SREBF1*, and *PPARγ* in the Sh1 and Sh2 cells compared to the NTC cells. The increase in the mRNA and protein expression of *FASN* along with the increased production of several long chain FAs in Sh1 and Sh2 cells indicated that fatty acid synthesis was enhanced in HSulf-1 silenced cells. To confirm, we also measured the enzymatic activity of FASN and found almost twofold higher activity of FASN in Sh1 and Sh2 cells compared to NTC cells (Figure 4D). These data suggest that loss of HSulf-1 can increase the activity of FASN to enhance FA synthesis.

Interestingly, FASN, SPHK1, PLA2G4A, PLA2G3, SREBF1, and PPARγ are the key enzymes which are localized and involved in the biogenesis of lipid droplets (LD) [50-54]. The FAs are activated by covalent modification by CoA via fatty-acyl-CoA synthetases and esterified to glycerol generating triglycerides or sterol esters that are stored in lipid droplets [42]. Accumulation of cytoplasmic LDs forms a basis of increased growth and chemoresistance in neoplastic cells [40,55]. To explore whether increased expression of above enzymes resulted in LD formation, we imaged the LDs by bodipy staining which clearly showed increased LDs in Sh1 and Sh2 cells. As expected, there were very few LDs in the NTC cells (Figure 4E). To confirm whether HSulf-1 was directly involved in LD biogenesis, we also rescued the expression of HSulf-1 in Sh1 cells by stable transfection of CMV-driven HSulf-1 expression construct (clone 11) with vector-transfected Sh1 cells served as controls (Figure 4F,G). Bodipy staining revealed that rescue of HSulf-1 significantly reduced the number of lipid droplets in Sh1 (Sh1 clone 11) compared to Sh1 vector control with many LDs (Figure 4G) demonstrating that loss of HSulf-1 promotes LD biogenesis. Additionally, transient transfection of PLA2G3 into OV202NTC cells showed increased LDs in these cells compared to vector-transfected controls (Figure 4H), suggesting a role of PLA2G3 in loss of HSulf-1 mediated LD biogenesis.

### Loss of HSulf-1 facilitates enhanced β-oxidation and lipolysis

**Figure 5:**
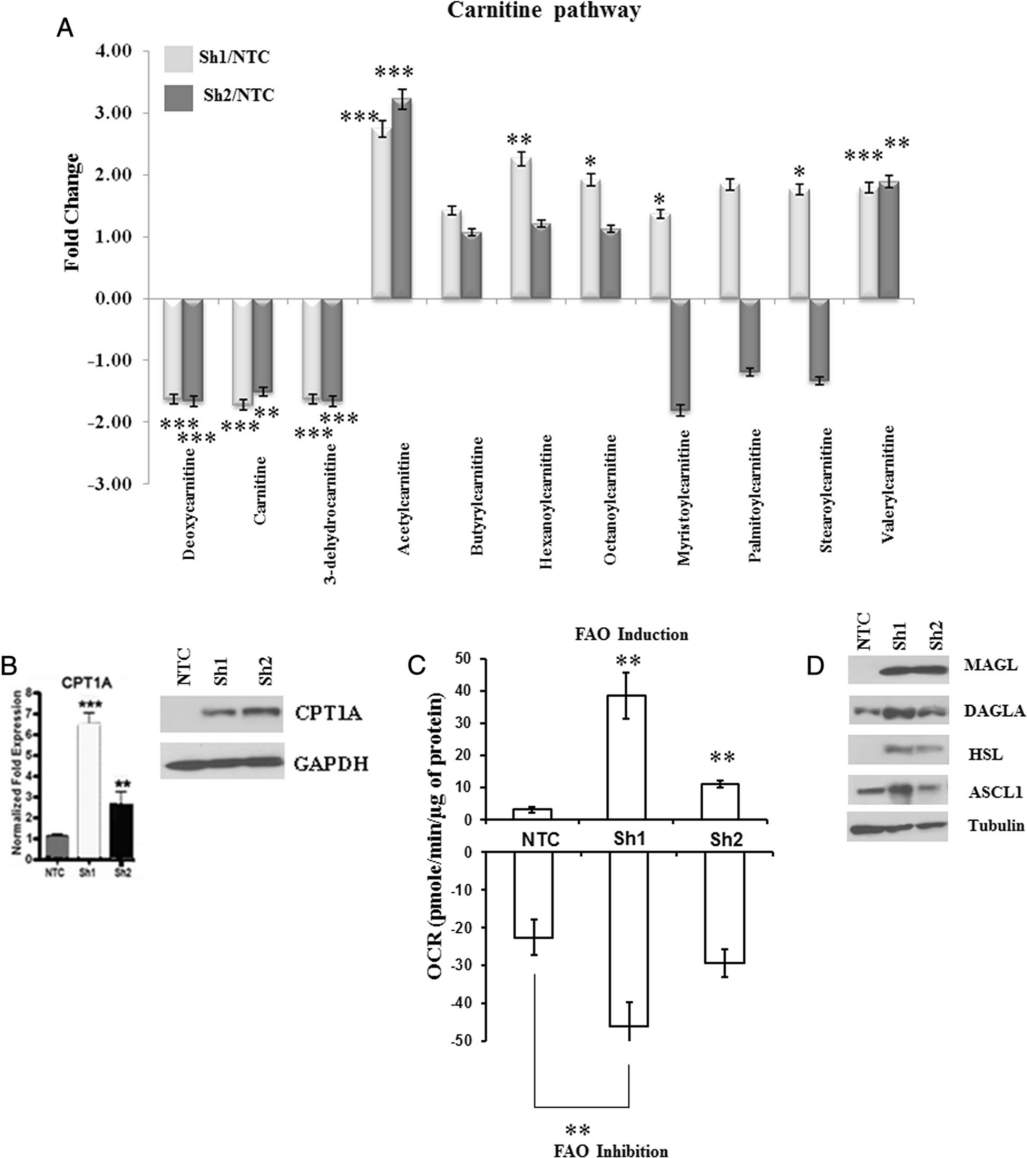
**Loss of HSulf-1 induced enhanced β-oxidation and lipolytic enzymes. (A)** The samples were extracted using Metabolon's standard solvent extraction method from cells in logarithmic phase having five biological replicates for each sample and distributed into equal parts for analysis on the GC/MS and LC/MS/MS platforms. Fold increase of carnitine and its derivatives were calculated by the average metabolite level of Sh1/NTC and Sh2/NTC. *p = 0.01 to 0.09; **p = 0.001 to 0.009; ***p < 0.001 compared to NTC. **(B)**. Levels of *CPT1A* in NTC, Sh1, and Sh2 cells determined by real-time PCR and Western blot analysis. *p < 0.05; **p < 0.01; ***p < 0.001 compared to NTC. **(C)** FAO in terms of OCR in pmol/min/mg of protein was monitored using a Seahorse Bioscience Extracellular Flux Analyzer in real time (mean ± S.D., *n* = 3). Cells treated with etomoxir (50 μM), an inhibitor of carnitine palmitoyltransferase 1, served as a positive control. Changes in the FAO induction in Sh1 and Sh2 cells are compared with that of NTC cells. Etomoxir-induced inhibition of FAO in Sh1 and Sh2 cells are compared with the FAO inhibition in NTC (*p* ≤ 0.01 and *p* ≤ 0.001). **(D)** Immunoblot analysis of MAGL, DAGLA, HSL, ASCL1 in NTC, Sh1, and Sh2 where tubulin used as internal loading control.

The analysis of metabolite data additionally revealed the increased levels of acetylcarnitine (FC = 3.74, *p = <0.001* and FC = 3.22, *p = <0.001*), butyrylcarnitine, hexanoylcarnitine and octanoylcarnitine in HSulf-1 silenced cells. Moreover, increased levels of oleolycarnitine were 16.84- and 6.64-fold in Sh1 and Sh2, respectively (Figure 5A). Carnitine is important for shuttling FAs across mitochondrial membranes for oxidation and so the escalation of carnitine level enhances β-oxidation support the increased growth of ovarian cancer. To determine if β-oxidation was enhanced in Sh1 and Sh2 cells, we measured the mRNA and protein level of carnitine palmitoyltransferase 1 (*CPT1A*) and the results showed a higher level of CPT1A in both in Sh1 and Sh2 cells (Figure 5B). The increase in the expression of *CPT1A* mRNA along with increased production of several of the long chain FAs in Sh1 and Sh2 cells indicated that these cells may utilize long chain FAs by β-oxidation to generate more ATP to accommodate increase proliferation. Next, to confirm this notion, we determined the fatty acid oxidation (FAO), and results showed that FAO was higher both in Sh1 and Sh2 compared to NTC cells upon addition of palmitate (Figure 5C). Subsequently, we demonstrated that Etomoxir, a specific inhibitor of CPT1A, decreased FAO more significantly in Sh1 and Sh2 cells compared to NTC cells. We also demonstrated that Sh1 and Sh2 cells were more sensitive toward Etomoxir treatment than NTC confirming a major role of FAO in their survival (Additional file 8: Figure S4).Since FAs are stored in lipid droplets and released by the action of lipolytic enzymes, we determined the expression of monoacylglycerol lipase (MAGL), diacylglycerol lipase alpha (DAGLA), long-chain acyl-CoA synthetase (ACSL1), and hormone-sensitive lipase (HSL). Immunoblot analysis showed that the expression of these enzymes was upregulated in Sh1 and Sh2 cells compared to NTC cells (Figure 5D), demonstrating that loss of HSulf-1 activates beta oxidation and lipolysis.

### Altered metabolism in amino acid and peptide super pathways in cells with loss of HSulf-1

In addition to the altered lipid metabolism, the metabolomic analysis revealed that amino acids and their derivatives were the second most class of metabolites altered in HSulf-1 knockdown cells (Additional file 4: Table S2). Most of the amino acids including serine, threonine, aspartate, asparagine, alanine, phenylalanine, tyrosine, tryptophan, arginine, ornithine, proline, and methionine were decreased in the range of -1.36- to -3.33-fold (*p* < 0.05) in both OV202 Sh1 and Sh2 cells. In contrast, glycine was increased marginally in Sh1 and Sh2 cells (range 1.08- to 1.1-fold; *p* < 0.001 for each). Interestingly, we found significant upregulation of cysteine (eight to tenfold, *p* < 0.001) and its derivatives, hypotaurine (FC = 2.38, *p = <0.001* and FC = 1.99, *p = <0.0244*) and taurine (FC = 9.91, *p = <0.0032* and FC = 5.38, *p = <0.0090*) in both Sh1 and Sh2 cells (Additional file 4: Table S2). We also observed alterations of *N*-acetylated amino acids including *N*-acetyl-alanine, serine, and threonine resulted from the action of *N*-acetyltransferases on acetyl-CoA and L-amino acids where all the six *N*-acetylated amino acid tested were downregulated. The amino acid-derived antioxidant, both reduced and oxidized glutathione levels were lower in HSulf-1-deficient cells. Additionally, among different dipeptide molecules, gamma-glutamylglutamate (FC = 3.73, 2.77 in Sh1 and Sh2, *p = <.0001*) and gamma-glutamylmethionine (FC = 7.31, 6.23 in Sh1 and Sh2 respectively, *p = <.0001*) levels were increased upon loss of HSulf-1. This enhancement may be particularly relevant as increased expression of gamma-glutamylglutamate is reported to be associated with tumor progression and drug resistance observed in human malignancies [56]. Additionally, a significant increase of pro-hydroxy-proline, (FC = 2.05, *p = 0.0057* and FC = 1.92, *p = 0.0088*) a dipeptide, also demonstrated in Sh1 and Sh2 cells. Pro-hydroxy-proline is a marker of collagen and extracellular matrix degradation (Additional file 4: Table S2).

## Discussion

Aberrant cellular metabolism in cancer is now well known and is directly related to tumorigenesis in most of the cancers [57,58]. Multiple signaling pathways and several molecules are involved in the synthesis and degradation of the lipids and also the activities of lipid metabolizing enzymes are regulated by a complex interplay between metabolic, tumor suppressor, and oncogenic signaling [59]. Though loss of HSulf-1, a putative tumor suppressor gene, was well known to promote tumorigenesis, angiogenesis [22,23], and invasion [20] in breast [18,21] and ovarian [23,24] cancers, the role of HSulf-1 was never elucidated in altered metabolism of ovarian cancer cells so far. Thus, in the present study, we have shown that loss of the putative tumor suppressor, HSulf-1 promotes altered metabolic pathways including lipid, amino acid, and nucleotide. Of the several pathways altered by loss of HSulf-1, the lipid and amino acid pathway-related metabolites accounted for 50% of the total altered metabolites identified. In ovarian cancer, altered lipid metabolism was detected in patients during early and late stages of disease compared to healthy controls [60,61]. In contrast to lipid pathway-related metabolites, there are very few reports on the alteration in the amino acid levels in OVCa. We found a significant downregulation of most of the amino acids with loss of HSulf-1 consistent with Zhang et al.'s report where they showed presence of lower levels of amino acids in the serum of patients with esophageal adenocarcinomas [62]. This downregulation could be due to differences in uptake from the media, increased utilization, and/or catabolism since increased demand for utilization of amino acids has been reported in other cancers [63,64]. Other studies have shown an increase in amino acid levels in the serum of patients with colon and breast cancers [65,66]. While the majority of amino acids were downregulated, we found significant upregulation of cysteine and its derivatives hypotaurine and taurine in Sh1 and Sh2 cells. Significant upregulation in cysteine, taurine, and hypotaurine could be the result of increased levels of cysteine dioxygenase type I (CDO1) [67-69] in Sh1 and Sh2 cells (data not shown). While taurine is reported to be downregulated in cancer [70], it is also reported to be increased in tumors of the prostate, squamous cell carcinoma and liver metastasis [71,72]. Additionally, levels of myo-inositol and taurine concentrations both *in vivo* and *in vitro* are correlated with cell density of the tumors [73].

Additionally, we also saw an increase in the dipeptide pro-hydroxy-pro in Sh1 and Sh2 cells. Increase in pro-hydroxy-proline is consistent with the report that major cartilage ECM proteins type II collagen and aggrecan were significantly lower in *HSulf*^*-*/-^ chondrocytes suggesting that loss of HSulf-1 may regulate the overall balance of cartilage matrix synthesis and degradation [74]. Consistent with this, significant alterations in several UDP-glycosylation moieties, including UDP-acetylglucosamine/UDP-acetylgalactosamine UDP-glucose, UDP-glucuronate, and UDP-galactose are also supportive of changes in extracellular matrix remodeling with reduced HSulf-1 expression.

The major pathway identified by both ingenuity pathway analysis and metabolic profiling was the alterations in the lipid pathway. Lipids are as important building blocks as carbohydrates to form the basic skeleton of rapidly dividing cells, and therefore, large amounts of FAs are required to accommodate high rates of proliferation in cancer cells [4]. Additionally, the source of FAs may determine the phospholipid composition of membranes. Moreover, it was reported that in high-grade ovarian cancer, long-chain fatty acids were elevated [75]. Our results indicate that with the loss of HSulf-1, there was a significant increase in the long-chain FAs along with branched and essential FAs in ovarian cancer. These high levels of FAs in ovarian cancer with the absence of HSulf-1 indicate a major role of HSulf-1 in FA synthesis. This enhanced production of FAs might meet the structural needs of highly proliferative ovarian cancer cells' requirement, when the tumor suppressor HSulf-1 is lost.

Sphingolipids and lysolipid or lysophospholipids are bioactive lipid molecules which play pivotal role in cancer pathogenesis. The most important sphingolipids are ceramide and sphingosine-1-phosphate and the balance between these two define the cell's fate. In cancer, sphingolipid metabolism is altered and includes changes in the levels of sphingolipids and the enzymes involved in their metabolism [76]. Lysolipids with their various FA side chains are also bioactive lipids which mainly function as growth-stimulating factor and induce cell proliferation, differentiation, and cell migration [77]. Herein, we report an increase in sphingolipids including sphinganine, sphingosine, palmitoylsphingomyelin along with altered choline/inositol and lysolipid content. Interestingly, according to the KEGG database, metabolite mapping and glycerophospholipid, arachidonic, and sphingolipid metabolisms were identified as key junctions. These results clearly indicate that with the loss of HSulf-1, the cell remodeled its own lipid synthesis in a manner to supply both structural and signaling lipids to malignant cells.

In cancer cells, the increased lipid synthesis is due to the higher expression and activity of lipogenic enzymes [78,79]. Changes in the expression and activity of enzymes involved in lipid metabolism are regulated by metabolic and oncogenic signalling pathways [80,81]. We identified enhanced expression of FASN, SREBF1, SPHK1, and PPARγ related to lipid biogenesis. Upregulation of these key lipogenic enzymes with the loss of HSulf-1 were further corroborated by their mRNA and protein expressions. Moreover higher enzymatic activity of FASN strongly supports the enhanced biogenesis of FAs with HSulf-1 loss. Additionally, phospholipase-related genes PLA2G3, PLA2G4A, PNPLA2, PLA2G4C, and PNPLA6 involved in lipolysis were also found to be higher in Sh1 and Sh2. Interestingly, PLA2G3 is an important enzyme in lipid metabolism and implicated in LD biogenesis [50,52]. Recently, it was reported that cancer cells contain increased numbers of lipid droplets compared with normal tissue [82] which are storage sites for triglycerides and cholesterol to be used as energy source. Loss of HSulf-1 induced an increase in PLA2G3 expression that may lead to the accumulation of LD in ovarian cancer. Furthermore, reduction in the number of LDs with the rescue of HSulf-1 expression in Sh1 cells establishes a direct connection to the loss of a putative tumor suppressor HSulf-1 and enhanced LD biogenesis in ovarian cancer.

In accordance with these results, we also demonstrated lower level of carnitine along with higher level of carnitine derivatives suggestive of enhanced transport of FAs through the mitochondrial membrane. More importantly, the augmented CPT1A level in Sh1 and Sh2 cells strongly support the possibility of enhanced β-oxidation. Increased and altered FA synthesis in cancer is a well-accepted phenomenon; however, enhanced expression and activity of lipolytic enzymes in tumor cells is a recent observation. In prostate cancer, malignant cells solely rely on FAO as their energy resource [83]. Here, we report that loss of HSulf-1 also promotes enhanced FAO in Sh1 and Sh2 cells compared to NTC. Consistent with increased lipolysis, our results indicated a significant increase in the expression of important lipolytic enzymes including MAGL, DAGLA, HSL, and ASCL1 in Sh1 and Sh2 cells compared to NTC cells. Higher MAGL expression has been shown to stimulate pro-tumorigenic signals and promotes survival, tumor growth, and migration [84]. In this regard, the relationship between synthesis, storage, and utilization of free fatty acids (FFA) through beta oxidation for energy production is very poorly understood. While the prevailing belief that cells that have increased FA synthesis usually do not undergo beta oxidation, it has also been suggested that newly synthesized FFA are immediately converted into neutral lipids and stored in lipid droplets [84]. The FFAs are released by the action of lipases such as mono-acyl glycerol (MAGL) which is then used for new membrane synthesis, lipid signaling and beta oxidation for energy production for the anabolic reactions. Our data seems to support this hypothesis since the Sh1/ Sh2 cells have both increased the rate of FA synthesis and beta oxidation compared to NTC cells. The FFA may be immediately converted to neutral lipids and stored in lipid droplets to be released under stress conditions.

Increasing evidence indicates that metabolic alterations induced by loss of tumor suppressor genes are common in cancer [16]. These alterations are critical for growth and survival of cancer cells. This is the first comprehensive report on metabolic alterations induced by loss of HSulf-1, a putative tumor suppressor gene in ovarian and breast cancer. Our results clearly indicate that loss of HSulf-1 remodels lipid metabolism in ovarian cancer. It is well accepted that HSulf-1 is a major regulator of growth factor-mediated signaling and altered the tumor microenvironment in ovarian cancer; however, how HSulf-1 directly reconstructs the lipid metabolism in terms of FA synthesis, lipolysis, and enhanced LD formation is yet to be unraveled. A better understanding of the function of HSulf-1, its role in energy metabolism (glycolysis, TCA cycles, PPP) and its regulation on target genes involved in altered cellular metabolism could lead to a better understanding of tumorigenesis as well as development of new targeted therapy.

## Conclusions

HSulf-1 is reported to be an important tumor-suppressor gene and its expression is lost in a majority of ovarian tumors. This study demonstrates a significant alteration of cellular metabolism upon loss of HSulf-1 in OV202 cells. Microarray analysis and metabolite profiling is performed to ascertain the impact of HSulf-1 on the overall metabolic changes including alterations in lipid and amino acid pathways. The major finding from our study shows the metabolic reprogramming of cells toward an enhanced lipid metabolism upon absence of HSulf-1. Cells adopt a lipogenic phenotype which is manifested with an excess fatty acid synthesis and an upregulated beta-oxidation. Furthermore, HSulf-1-deficient cells accumulate a huge amount of cytoplasmic lipid droplets to accommodate the excessive fatty acid syntheses. The present findings are supported by the increasing evidences of enhanced lipid metabolism in cancer cells facilitating cell survival, proliferation, and signaling. Our results indicate that, loss of HSulf-1 is enabling the cells to synthesize more lipids to expedite the high proliferation rate and survival.

## Abbreviations

ACSL1: long-chain acyl-CoA synthetase; CPT1A: carnitine palmitoyltransferase 1; DAGLA: diacylglycerol lipase alpha; FDR: false discovery rate; FA: fatty acid; FAO: fatty acid oxidation; FASN: fatty acid synthase; GC/MS: LC/MS/MS: PCA: principal component analysis; HSL: hormone sensitive lipase; KEGG: Kyoto Encyclopedia of Genes and Genomics; LD: lipid droplet; MAGL: monoacylglycerol lipase; NTC: OV202: non-targeted control cells; PPARγ: peroxisome proliferator-activated receptor gamma; SAM: significance analysis of microarray; Sh1: stably knockdown HSulf-1 clonal line 1 in OV202; Sh2: stably knockdown HSulf-1 clonal line 2 in OV202; SPHK1: sphingosine kinase 1; SREBF1: sterol regulatory element-binding transcription factor 1.

## Competing interests

EDK and LNB are employees of Metabolon, Inc. and, as such, have affiliations with or financial involvement with Metabolon, Inc. These authors have no other relevant affiliations or financial involvement with any organization or entity with a financial interest in or financial conflict with the subject matter or materials discussed in the manuscript apart from those disclosed. The rest of the authors declare that they have no competing interests.

## Authors’ contributions

DR technically performed the experiments in Figures 4C,E,G,H and 5B,C,D. He also wrote portions of the manuscript and the ‘Methods’ section for FASN and beta oxidation section and was involved in the discussions. SM wrote a major part of the Introduction and Results and discussion, provided figures related to lipid metabolites, western blots in Figure 2E, and performed beta oxidation-related studies in Figure 5C. CW did the bioinformatics analysis of the KEGG pathway of the metabolites, the ingenuity pathway analysis of the microarray data and provided the following figures: Figure 1B,D, Additional file 3: Figure S2, Figure 2, Additional file 7: Figure S3, and wrote the corresponding section in the MS. XH generated the OV202NTC, Sh1, and Sh2 cells and the rescue clone Sh1 Cl11. AK performed the FASN activity assay and edited the manuscript. SG edited the manuscript and provided input regarding the metaboanalyst analysis. RH and DBJ extracted RNA and provided cDNAs for real-time PCR analysis. SK edited the manuscript and provided financial support for visiting student DBJ. EC and JCP were involved in the discussions and editing of the manuscript. CF trained DR and SM in the use of Seahorse-related experiments for beta oxidation. AM, SD, and JBG edited the manuscript and provided funds to support a technician involved in the study. SR and AO designed the microarray study and normalized the microarray data, provided the write-up related to these methods in the ‘Methods’ section, and reviewed the final manuscript. EDK edited the manuscript. LNB provided the initial write-up on the analysis of the overall metabolomics data and edited the manuscript. JC did the SAM analysis and was involved in the discussion and editing of the manuscript. VS was involved in the design, execution and analysis and did the QPCR of the lipid-related genes and wrote the manuscript with SM and DR. All authors read and approved the final manuscript.

## Supplementary Material

Additional file 1: Figure S1 Loss of HSulf-1 induced enhanced proliferation in OV202 cells. Equal number of cells of NTC, Sh1, and Sh2 were plated in triplicates (1 × 10^5^) and counted after 24, 48, and 72 h. The increase in cell count in both Sh1 and Sh2 were statistically significant in 48 h (*p < 0.05) and 72 hr (**p < 0.001). These experiments were repeated twice. Click here for file

Additional file 2: Table S1 Microarray analyses of OV202 cells upon HSulf-1 loss. Click here for file

Additional file 3: Figure S2 Multivariate regression results between major metabolite classes. The eight major metabolite classes are amino acid, carbohydrate, cofactors and vitamins, energy, lipid, nucleotide, peptide, and xenobiotics. ‘Estimate’ and ‘Std. Error’ are estimate of regression coefficient and estimation of standard-deviation error; ‘*t* value’ is the *t* statistics for each coefficient estimate, and ‘Pr(>|t|)’ is the corresponding *p* value for each coefficient estimate. Click here for file

Additional file 4: Table S2 Changes in major metabolic pathways including amino acids, lipids and nucleotides. List of metabolites identified through mass spectrometry and the super-pathway and sub-pathway for them are shown. The green- and red-shaded boxes are metabolites that are downregulated and/or upregulated in Sh1 and Sh2 cells compared to NTC cells respectively. The *p* values and the platform used to identify these metabolites are also shown. Click here for file

Additional file 5: Table S3 Global metabolic changes upon HSulf-1 loss. List of significantly altered metabolites in Sh1 and Sh2 cells compared to NTC cells in different pathways (XLS). Heat map of statistically significant biochemical profiles in this study. By paired comparisons, shaded cells indicate *p* ≤ 0.05 (red indicates that the mean values are significantly higher compared untreated control; green values significantly lower). Blue-bolded text indicates 0.05 < *p* < 0.10. All data were normalized using Bradford protein concentration (red—upregulated, green—downregulated, and blue—approaching significance). Click here for file

Additional file 6: Table S4 Alterations in lipid metabolites. Lipid metabolic pathways in Sh1 and Sh2 cells compared to NTC cells. Heat map of statistically significant (*p* ≤ 0.05, FDR < 0.05) biochemicals are shown. Red indicates that the mean values are significantly higher by comparison with untreated control; green values significantly lower. All data were normalized using Bradford protein concentration (red—upregulated, green—downregulated. Click here for file

Additional file 7: Figure S3 Metaboanalyst pathway analysis. **(A)** Statistics for pathways with major change based on high impact (linoleic acid metabolism) or *p* value (pathways glycerophospholipid, arachidonic, and sphingolipid metabolic pathways). **(B)** Of the 12 highly significant KEGG pathways plotted according to global test *p* value (intensity of color in the vertical axis) and impact factor (size of the circles in the horizontal axis), all 4 belong to the lipid pathways. Click here for file

Additional file 8: Figure S4 Effect of etomoxir on cellular growth with increasing concentration of Etomoxir treatment (0 to 100 μM) in NTC, Sh1, and Sh2 cells (*n* = 2). At 60 μM and onwards, the cell growth inhibition was statistically significant (*p* < 0.05) in both Sh1 and Sh2 cells compared to NTC. Click here for file
