# Erratum to: Loss of HSulf-1 promotes altered lipid metabolism in ovarian cancer

**DOI:** 10.1186/2049-3002-2-24

**Published:** 2014-11-04

**Authors:** Debarshi Roy, Susmita Mondal, Chen Wang, Xiaoping He, Ashwani Khurana, Shailendra Giri, Robert Hoffmann, Deok-Beom Jung, Sung H Kim, Eduardo N Chini, Juliana Camacho Periera, Clifford D Folmes, Andrea Mariani, Sean C Dowdy, Jamie N Bakkum-Gamez, Shaun M Riska, Ann L Oberg, Edward D Karoly, Lauren N Bell, Jeremy Chien, Viji Shridhar

**Affiliations:** Department of Experimental Pathology, Mayo Clinic College of Medicine, Rochester, MN 55905 USA; Division of Biomedical Statistics and Informatics, Mayo Clinic, Rochester, MN 55905 USA; Henry Ford Health System, Detroit, MI 48202 USA; Cancer Preventive Material Development Research Center (CPMRC), College of Oriental Medicine, Kyunghee University, Seoul, 130-701 Republic of Korea; Department of Anesthesiology, Mayo Clinic College of Medicine, Rochester, MN 55905 USA; Department of Cardiovascular Disease, Mayo Clinic College of Medicine, Rochester, MN 55905 USA; Department of Obstetrics and Gynecology, Mayo Clinic College of Medicine, Rochester, MN 55905 USA; Metabolon, Inc, Durham, NC 27713 USA; Department of Cancer Biology, University of Kansas Medical Center, Kansas City, KN 66160 USA

## Correction

After publication of this manuscript [1], it has been brought to our attention that we have on several occasions referred to the long-chain acyl-CoA synthetase as ASCL1. In all such cases, the correct abbreviation of ACSL1 should have been used.
