# Supplementary figures and images for: Loss of HSulf-1 promotes altered lipid metabolism in ovarian cancer
Source: Cancer Metab. 2014 Aug 18;2:13. doi: 10.1186/2049-3002-2-13 (PMC4164348; doi:10.1186/2049-3002-2-13)

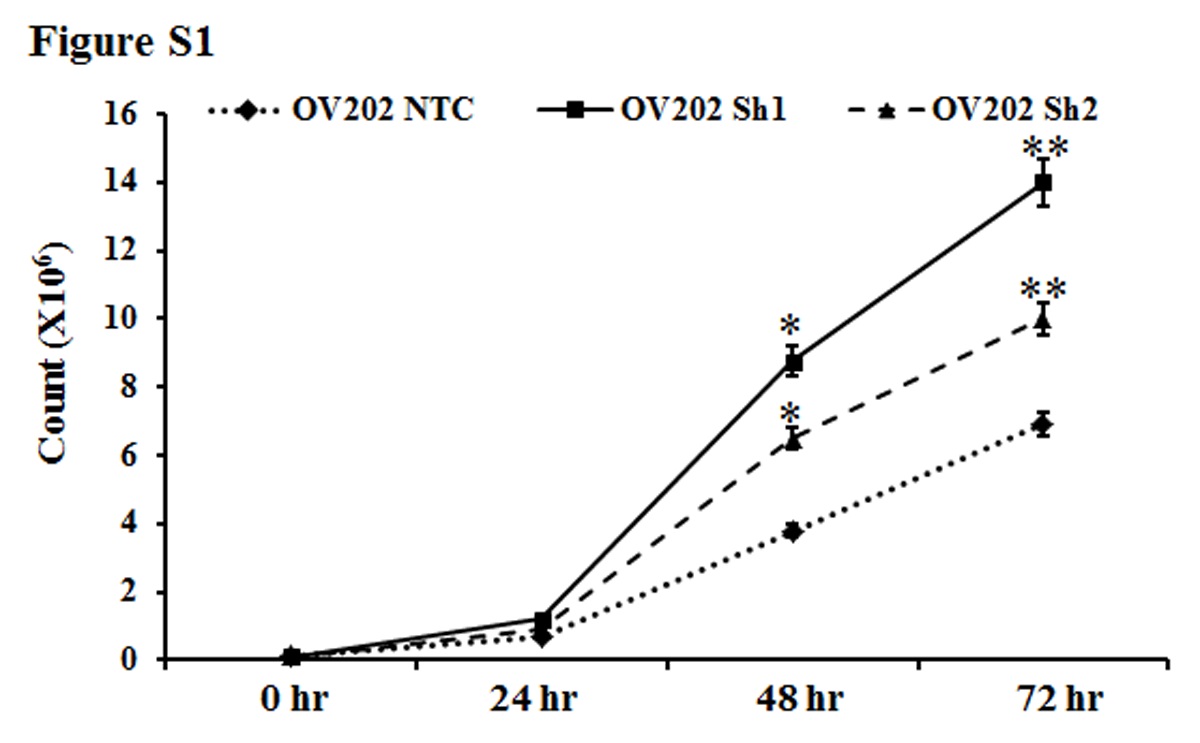

Supplement: Additional file 1: Figure S1 — Loss of HSulf-1 induced enhanced proliferation in OV202 cells. Equal number of cells of NTC, Sh1, and Sh2 were plated in triplicates (1 × 10^5^) and counted after 24, 48, and 72 h. The increase in cell count in both Sh1 and Sh2 were statistically significant in 48 h (*p < 0.05) and 72 hr (**p < 0.001). These experiments were repeated twice. [file 2049-3002-2-13-S1.tiff]

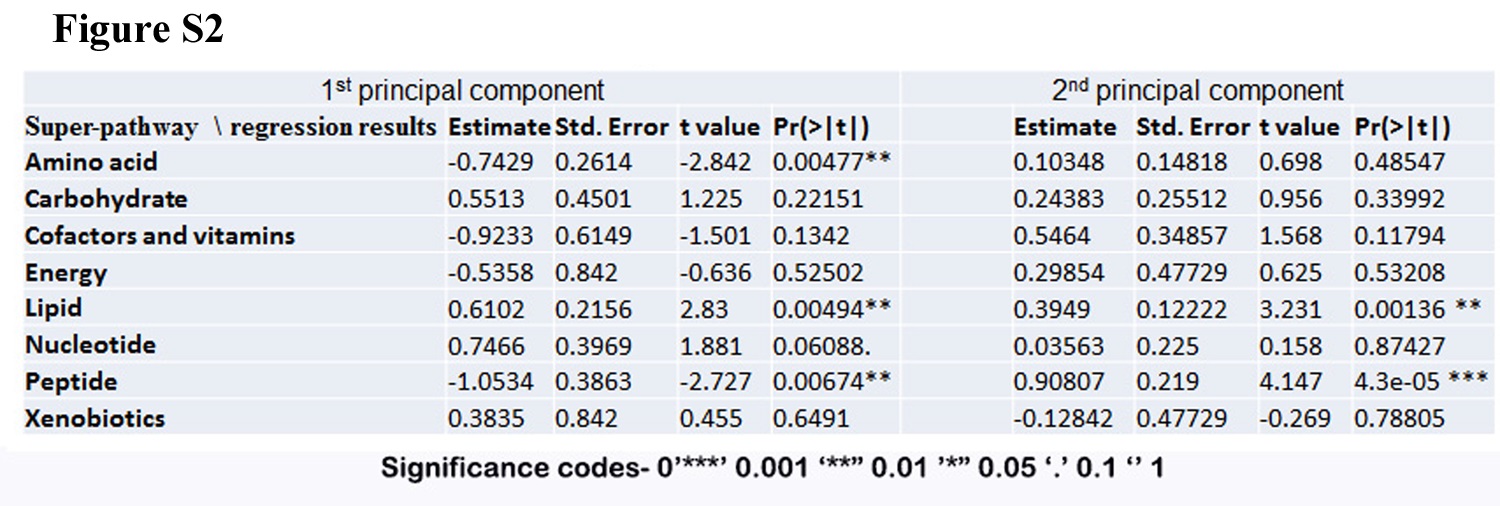

Supplement: Additional file 3: Figure S2 — Multivariate regression results between major metabolite classes. The eight major metabolite classes are amino acid, carbohydrate, cofactors and vitamins, energy, lipid, nucleotide, peptide, and xenobiotics. ‘Estimate’ and ‘Std. Error’ are estimate of regression coefficient and estimation of standard-deviation error; ‘t value’ is the t statistics for each coefficient estimate, and ‘Pr(>|t|)’ is the corresponding p value for each coefficient estimate. [file 2049-3002-2-13-S3.tiff]

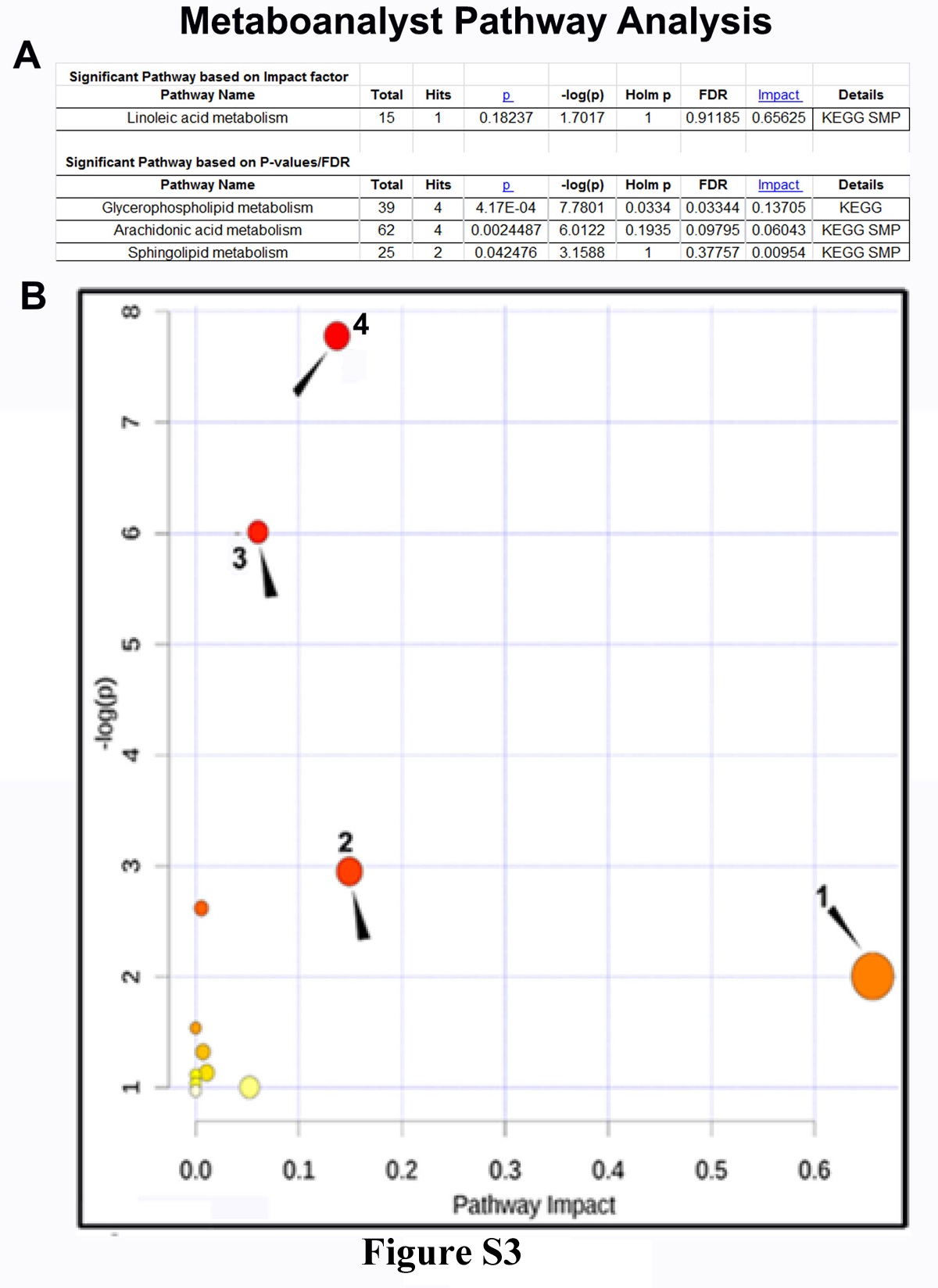

Supplement: Additional file 7: Figure S3 — Metaboanalyst pathway analysis. (A) Statistics for pathways with major change based on high impact (linoleic acid metabolism) or p value (pathways glycerophospholipid, arachidonic, and sphingolipid metabolic pathways). (B) Of the 12 highly significant KEGG pathways plotted according to global test p value (intensity of color in the vertical axis) and impact factor (size of the circles in the horizontal axis), all 4 belong to the lipid pathways. [file 2049-3002-2-13-S7.tiff]

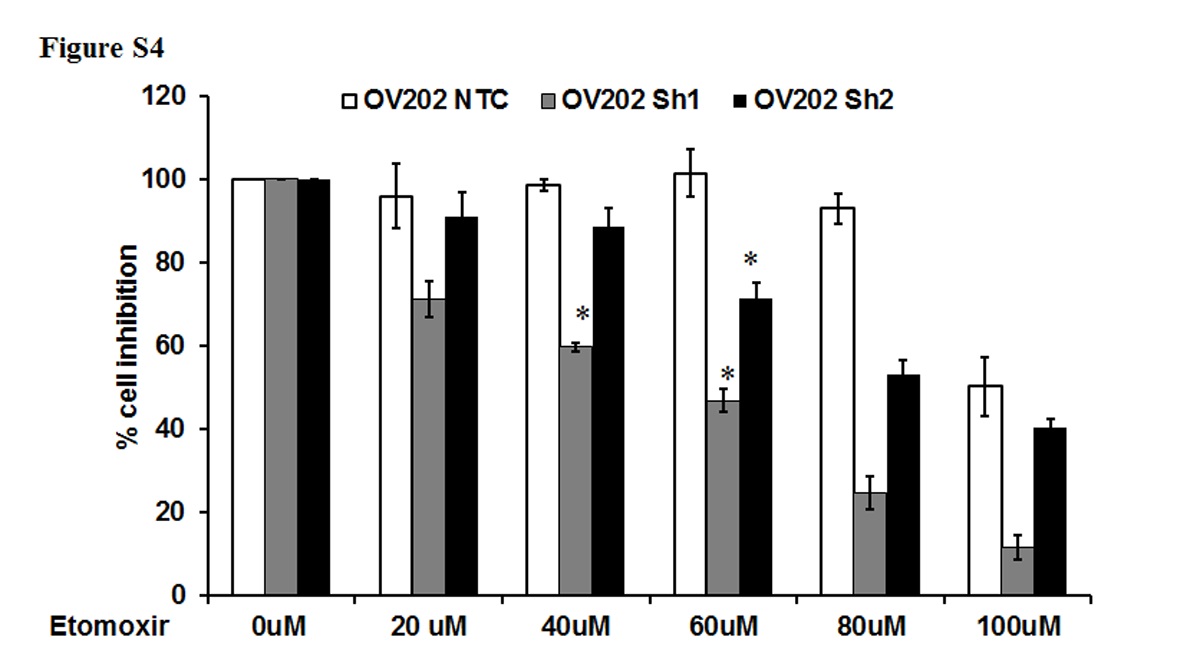

Supplement: Additional file 8: Figure S4 — Effect of etomoxir on cellular growth with increasing concentration of Etomoxir treatment (0 to 100 μM) in NTC, Sh1, and Sh2 cells (n = 2). At 60 μM and onwards, the cell growth inhibition was statistically significant (p < 0.05) in both Sh1 and Sh2 cells compared to NTC. [file 2049-3002-2-13-S8.tiff]
